# Calorimetric Studies of Magnesium-Rich Mg-Pd Alloys

**DOI:** 10.3390/ma14030680

**Published:** 2021-02-02

**Authors:** Adam Dębski, Sylwia Terlicka, Władysław Gąsior, Wojciech Gierlotka, Magda Pęska, Julita Dworecka-Wójcik, Marek Polański

**Affiliations:** 1 Institute of Metallurgy and Materials Science, Polish Academy of Sciences, 25 Reymonta Street, 30-059 Cracow, Poland; a.debski@imim.pl (A.D.); s.terlicka@imim.pl (S.T.); w.gasior@imim.pl (W.G.); 2 Department of Materials Science and Engineering, National Dong Hwa University, Shoufong 974, Taiwan; wojtek@gms.ndhu.edu.tw; 3 Department of Functional Materials and Hydrogen Technology, Military University of Technology, 2 Kaliskiego St., 00-908 Warsaw, Poland; magda.peska@wat.edu.pl (M.P.); julita.dworecka@wat.edu.pl (J.D.-W.)

**Keywords:** formation enthalpy, drop calorimetry, solution in aluminum bath, Mg-Pd alloys

## Abstract

Solution calorimetry with liquid aluminum as the bath was conducted to measure the enthalpy of a solution of magnesium and palladium as well as the standard formation enthalpies of selected magnesium-palladium alloys. These alloys were synthesized from pure elements, which were melted in a resistance furnace that was placed in a glove box containing high-purity argon and a very low concentration of impurities, such as oxygen and water vapor. A Setaram MHTC 96 Line evo drop calorimeter was used to determine the energetic effects of the solution. The enthalpies of the Mg and Pd solutions in liquid aluminum were measured at 1033 K, and they equaled −8.6 ± 1.1 and −186.8 ± 1.1 kJ/mol, respectively. The values of the standard formation enthalpy of the investigated alloys with concentrations close to the Mg_6_Pd, ε, Mg_5_Pd_2_, and Mg_2_Pd intermetallic phases were determined as follows: −28.0 ± 1.2 kJ/mol of atoms, −32.6 ± 1.6 kJ/mol of atoms, −46.8 ± 1.4 kJ/mol of atoms, and −56.0 ± 1.6 kJ/mol of atoms, respectively. The latter data were compared with existing experimental and theoretical data from the literature along with data calculated using the Miedema model.

## 1. Introduction

Energy is a very important commodity in life. Most energy still comes from natural sources, such as coal and oil, but scientists all over the world are constantly searching for an alternative, renewable, and efficient energy source to reduce the climate change caused by the combustion products of natural fuels, which have a negative impact on the climate [1]. Hydrogen is the best-known chemical energy carrier that can be very effectively converted to electricity in Proton-Exchange Membrane Fuel Cells with only water and heat generation. The main problem scientists are trying to solve is finding a suitable material for hydrogen storage with the possibility of the fast absorption and desorption of hydrogen, especially in applications for mobile devices [2,3,4].

Research on solid-state hydrogen storage materials has been conducted for many years. Some of these materials are metals and their alloys and are capable of reversibly absorbing large amounts of hydrogen. Magnesium has been studied extensively for applications as a hydrogen storage material because magnesium hydride, which Mg creates as it reacts with hydrogen, has a high gravimetric and volumetric density of hydrogen storage (7.6 mass % and 110 g H/L, respectively) [5,6,7]. However, its high enthalpy of decomposition requires high operating temperatures for the desorption of hydrogen, while the slow diffusion kinetics of hydrogen by mass, for example, poses challenges for its large-scale deployment. To overcome these difficulties, small amounts of additives are added to magnesium to create magnesium compounds, which, in relation to pure Mg, improves the unfavorable thermodynamics and sometimes the kinetics of the reaction [8]. Significant improvements were made in this field in order to modify the thermodynamics of Mg-based systems starting more than 50 years ago [9,10], but the research is in this area is continuing, including alloying with transition [11,12,13,14,15,16,17] catalysts [18,19], complex hydride additives [20], and even mechanical processing [21,22,23]. It has been indicated that the addition of noble metals, such as palladium or silver, can enhance the storage properties of magnesium [16,24,25,26,27]. Despite this, the thermodynamics and phase diagrams for magnesium systems such as Mg-Pd and Mg-Pt (and others) are limited and sometimes incomplete; this knowledge is necessary for designing and producing proper materials.

The phase diagram of the Mg-Pd system was estimated for the first time by Nayeb-Hashemi and Clark [28]. It was based on limited data presented by [29,30,31] and contained five uncertain intermetallic phases (Mg_6_Pd, Mg_4_Pd, Mg_5_Pd_2_, MgPd, and Mg_0.9_Pd_1.1_).

**Figure 1:**
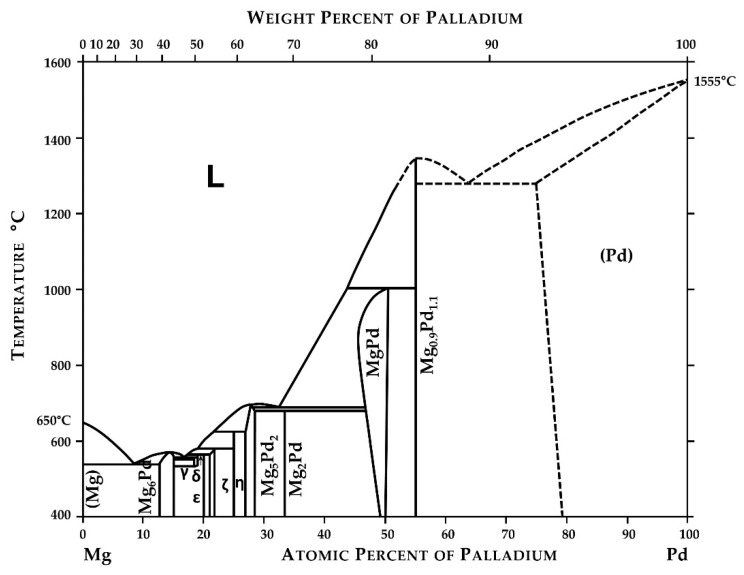
Phase diagram of the Mg-Pd system. Reprinted with permission from ref. [33]. 2010, Springer Nature.

Next, based on their own experimental data from differential thermal analysis (DTA) for Pd alloys for the composition range between 0 and 56 at.%, Makongo et al. [32] presented a new variant of the binary system which was quite different from what Nayeb-Hashemi and Clark [28] proposed. The last version of the Mg-Pd system was published by Okamoto [33] and is reproduced in Figure 1.

The calculations of the formation energy for the Mg_6_Pd, Mg_57_Pd_13_, Mg_3_Pd, Mg_5_Pd_2_, and MgPd intermetallic phases were conducted and published by Fernandez et al. [34,35]. The first experimental values of the formation enthalpy of the Mg_6_Pd and Mg_5_Pd_2_ intermetallic compounds were measured by Delsante et al. [36] using direct drop calorimetry, and they were equal to −28.2 ± 1.0 and −39.5 ± 2.8 kJ/mol of atoms, respectively. The formation enthalpies of six Mg-rich alloys corresponding to intermetallic phases from the Mg-Pd system were also presented in our previous work [37]. They were investigated using solution calorimetry in a liquid tin bath, and the determined formation energies equaled −27.0 ± 0.8, −34.4 ± 0.9, −35.2 ± 1.4, −44.2 ± 0.9, −46.0 ± 0.7, and −54.3 ± 2.3 kJ/mol of atoms for alloys containing 14.6 at.% Pd, 19.4 at.% Pd, 20.1 at.% Pd, 27.7 at.% Pd, 29.3 at.% Pd, and 35.5 at.% Pd, respectively. Moreover, the ab initio calculations of the formation energies of all existing intermetallic phases shown in Figure 1 were also reported in our other work [38].

This work is a continuation of research on the thermodynamic properties of the Mg-Pd system initiated by our group. This paper presents an extension of the results of the formation enthalpies of Mg-rich alloys which correspond to intermetallic phases. During the calorimetric measurements, different types of metallic baths were used. The choice of bath is determined by its ability to dissolve the test components forming the alloy during the test. In addition, the bath should have a low melting point, negligible evaporation pressure in the temperature range chosen for tests, and a lower density (as compared to the tested specimen) to prevent the sample from floating on the surface of the liquid bath. For many years, molten tin was used as the main solvent in the calorimetric measurements. However, liquid tin was not always is the best possible solvent for dissolving transition metals, as discussed by Colinet in [39]. In the case of the mentioned research group, liquid aluminum is often used. Despite the fact that the formation enthalpy is a physical value and theoretically should not be affected by the bath type, in practice the measured value is affected by measurement conditions. For these reasons, the presented investigations were conducted by solution calorimetry using a liquid aluminum bath in order to compare the obtained results with previous measurements.

## 2. Materials and Methods

Table 1 contains a list of the materials that were applied to determine the standard enthalpies of the formation of the investigated alloys. These alloys were prepared in an glove box (Labmaster, MBraun, Garching, Germany) in a high-purity argon atmosphere (H_2_O < 0.5 ppm, O_2_ < 0.1 ppm, N_2_ was not monitored and was absorbed by Ti at 1100 K). Calculated and weighted (0.1 mg precision) amounts of metals (Pd and Mg) were melted in a resistance furnace in stainless steel crucibles (AISI 304L, Accelor Mittal, Luxembourg). After melting and careful stirring, the liquid alloys were poured into a specially designed steel ingot mold. Finally, the obtained alloys were annealed at 663 K for 72 and 84 h (Table 2) in the furnace that was placed in the glove box containing the protective atmosphere characterized above.

The structural studies of the presented Mg-Pd alloys were conducted after the homogenization process with the use of X-ray diffraction (Ultima IV; Rigaku, Tokyo, Japan; Co Kα radiation source; 1.79026 Å) and SEM/EDS (FEI Quanta 3D SEM). A full description of these results was presented in our previous work [37], and both the results of phase analyses and SEM observations are shown in the Supplementary Materials, Figures S1–S4.

The calorimetric studies were performed in a protective argon atmosphere with the use of a Setaram MHTC 96 line evo drop calorimeter using alumina crucibles. The conducted calorimetric studies were similar to our previous calorimetric measurements presented in [40,41,42]. Before each experiment began, the workspace of the calorimeter was purified by evacuation with a vacuum pump and flushed with high-purity argon. Next, the calibration constant was determined using six pieces of Al.

The enthalpy of the formation (Δ_f_*H*) values of the measured phases at 298 K were calculated from the difference in the heat effects, which corresponded to heating the samples from room temperature (298 K) to the measurement temperature (1033 K) and observing the dissolution of the studied phases and their components in the aluminum bath. The Δ_f_*H* values were computed using the following equation:

(1) $$\Delta_{f}H=x_{\mathrm{Mg}}\Delta H_{\mathrm{Mg}}^{0}+x_{\mathrm{Pd}}\Delta H_{\mathrm{Pd}}^{0}-\Delta H_{x_{\mathrm{Mg}}x_{\mathrm{Pd}}}^{0}$$

where Δ_f_*H* is the enthalpy of the formation of the measured phase; *x*_Mg_ and *x*_Pd_ are the mole fractions of the components, respectively; and $\Delta H_{\mathrm{Mg}}^{0}$, $\Delta H_{\mathrm{Pd}}^{0}$, and $\Delta H_{x_{\mathrm{Mg}}x_{\mathrm{Pd}}}^{0}$ are the heat effects accompanying the dissolution of one mole of the components (Mg and Pd) and phases in the aluminum bath, respectively. The $\Delta H_{\mathrm{Mg}}^{0}$ and $\Delta H_{\mathrm{Pd}}^{0}$ values are the sums of the limiting partial enthalpy of the solution of liquid Mg and Pd in a liquid Al bath and the enthalpy change of the pure Mg and Pd from room temperature to measurement temperature:

(2) $$\Delta H_{\mathrm{Mg}}^{0}=\Delta_{\mathrm{sol}}{\bar{H}}_{\mathrm{Mg}\left(l\right)}^{\infty }+\Delta H_{\mathrm{Mg}}^{T_{298}\to T_{1033}}$$

(3) $$\Delta H_{\mathrm{Pd}}^{0}=\Delta_{\mathrm{sol}}{\bar{H}}_{\mathrm{Pd}\left(l\right)}^{\infty }+\Delta H_{\mathrm{Pd}}^{T_{298}\to T_{1033}}$$

In this study, the heat effects $\Delta H^{\mathrm{ef}}$ of the dissolution of the binary alloys as well as metals were measured.

## 3. Results and Discussion

The limiting partial enthalpy of the solution of Mg and Pd in liquid aluminum was measured at the first stage of the calorimetric investigations. The necessary thermochemical data of metals were calculated using Pandat 2013 [43] (Pan SGTE database based on the original SGTE v4.4 database [44]). The experimental results of the limiting partial enthalpy of the solution of Mg and Pd in liquid aluminum are presented in Table 3 and Table 4, respectively.

The standard enthalpies of the formation of the Mg-Pd alloys were determined by employing solution calorimetry. The obtained results are presented in Table 5 together with the standard errors.

**Figure 2:**
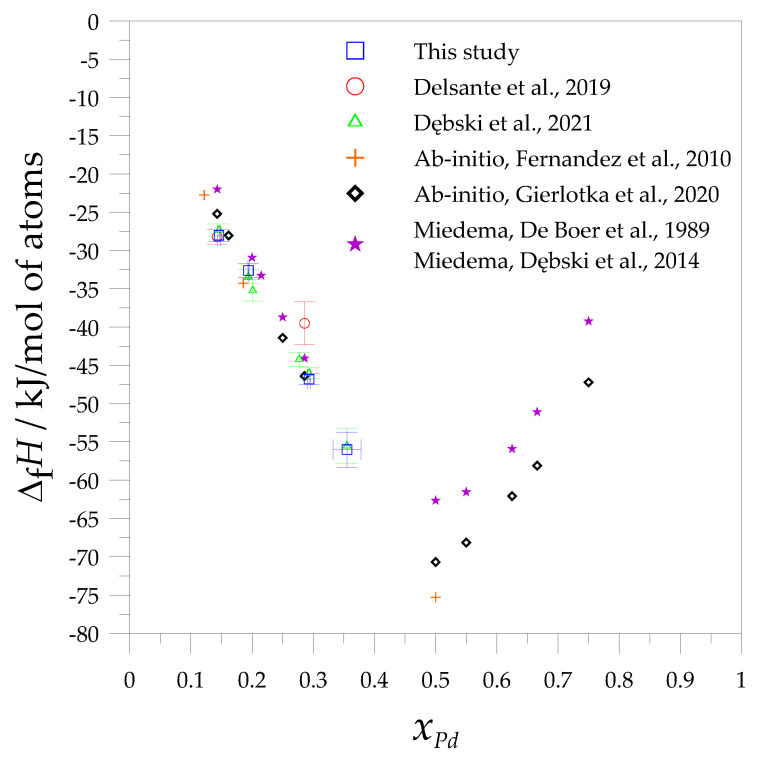
Comparison of the experimental and calculated values of the standard formation enthalpies of the Mg-Pd intermetallic phases and alloys (solution method: in liquid aluminum—this study, in liquid tin (Dębski et al., 2021 [37]), direct reaction method (Delsante et al., 2019 [36]), with ab initio calculations (Fernandez et al., 2010 [34]; Gierlotka et al., 2020 [38]) and the Miedema model (De Boer et al., 1989 [45]; Dębski et al., 2014 [46]).

The comparison of the formation enthalpies of the investigated alloys obtained in this study is presented in Figure 2, together with the experimental data obtained from the direct reaction method [36], as well as the results from the ab initio method [34,38] and calculations using the Miedema model [45,46].

As seen in Figure 2, the addition of palladium affects the lowering of the enthalpy of the formation values of the studied alloys. This trend is observed to *x*_Pd_ = 0.5, which is also documented by the Miedema model and ab initio calculations. Moreover, the obtained formation enthalpy of the alloy close to the composition of the Mg_6_Pd intermetallic phase is in very good agreement with the data measured by the direct reaction method [36], as well as the ab initio calculations [38]. A large discrepancy is observed between the values calculated using the Miedema model [45,46] and the experimental measurements, which reach ~6 kJ/mol of atoms. In the case of the enthalpy of the formation of the alloy in which the concentration is close to the ε- intermetallic phase, the results obtained from the calculations are similar to those obtained from the measurements. In regard to alloys close to the Mg_5_Pd_2_ phase, the greatest differences in values are observed between those measured by the direct reaction method and those obtained by the solution method (in Al and Sn), and these calculated values fluctuate between 5 and 7 kJ/mol of atoms. One can suppose that several reasons influence the discrepancy between the results from the direct synthesis method and those obtained from the solution method. In the direct reaction method, the reason for this may be the partial reaction of the sample during the preparation of the powders, the oxidation of the powders, and the fact that the reaction in the calorimeter may not be complete during the measurement. Moreover, for the direct method, the XRD studies were performed after the sample had cooled down together with the calorimeter, which allowed the sample to have a longer reaction time. Taking these factors into consideration for the discrepancies obtained with the Mg_5_Pd_2_ phase, it seems that the dissolution method appears to be more accurate for measuring the remaining palladium-rich phases.

Similar observations have been reported by Rzyman et al. [47], who compared the enthalpies of the formation of intermetallic phases from the Al-Ti system obtained by the direct reaction and solution calorimetric methods. Only in the case of the Al_3_Ti phase were the results obtained from both calorimetric methods in good agreement, while for the remaining phases from the Al-Ti system the differences were about 5 kJ/mol of atoms for the AlTi phase and about 10 kJ/mol of atoms for the AlTi_3_ phase. Moreover, it was proven that, during the reaction of titanium and aluminum powders, the first obtained product was the Al_3_Ti phase, regardless of the applied proportion. For this reason, the data for the enthalpy of formation for the Al_3_Ti phase obtained from both methods are consistent. In the case of the AlTi and AlTi_3_ phases, the observed differences in the enthalpy of formation values indicate that the reaction of phase formation was not completed in the calorimeter, and this is the reason for the differences in the results obtained from the two methods.

## 4. Conclusions

This paper presents experimental data of the limiting partial enthalpy of a solution of magnesium and palladium in liquid aluminum at 1033 K, as well as the standard formation enthalpy values of four alloys with chemical compositions close to the Mg_6_Pd, ε, Mg_5_Pd_2_, and Mg_2_Pd intermetallic phases that were measured by solution calorimetry a liquid aluminum bath. The obtained data for the limiting partial enthalpy of a solution of Pd and Mg in liquid aluminum can be used in future studies of phases and alloys containing these metals in their composition.

The obtained value for the formation enthalpy of the alloy close to the Mg_6_Pd intermetallic phase agrees well with both the values obtained by the solution calorimetry in liquid Sn and direct reaction methods.

In the case of an alloy with a composition very close to the Mg_5_Pd_2_ intermetallic phase, the Δ_f_*H* values determined by both solution calorimetry methods are similar and more exothermic than the data obtained by the direct reaction method.

Moreover, data on the standard enthalpies of formation of the Mg-Pd solid phases measured by solution calorimetry showed a slightly better correlation with those obtained by the ab initio calculations than those calculated by the Miedema model.

The calculated formation enthalpies of the Mg-Pd phases and alloys by the ab initio method were more exothermic in comparison to those calculated by the Miedema model, and the observed differences varied between 5 and 15 kJ/mol of atoms.

## Figures and Tables

**Table 1:** Specifications of the applied materials.

Chemical Name	Source	Purity (Mass %)	Analysis Method
Magnesium	Sigma Aldrich	99.9	Certified purity
Palladium	Safina a.s.	99.95	Certified purity
Argon	Air Products	99.9999	Certified purity

**Table 2:** Homogenization conditions of the prepared alloys.

No.	Alloys (Phases)	Annealing Temperature	Annealing Time (h)
1	14.6 at.% Pd	663	84
2	19.4 at.% Pd	663	84
3	29.3 at.% Pd	663	72
4	35.5 at.% Pd	663	72

**Table 3:** Values of the limiting partial enthalpy of the solution of liquid Mg $\Delta_{\mathrm{sol}}{\bar{H}}_{\mathrm{Mg}\left(l\right)}^{\infty }$ in liquid Al. Atmosphere: argon at a pressure *p* = 0.1 MPa; calibration constant *K* = 0.000003207 kJ/μVs; enthalpy of the pure Mg $\Delta H_{\mathrm{Mg}}^{T_{298}\to T_{1033}}$ = 30.0048 kJ/mol; temperature of the Al bath *T_M_* = 1033 K; and drop temperature *T_D_* = 298 K.

Measurement No.	Dropped Mass of Samples (g)	At.% of Mg in Al Bath	Heat Effects Δ*H*^ef^ (kJ/mol)	$\mathrm{Limiting}\;\mathrm{Partial}\;\mathrm{Enthalpy}\;\mathrm{of}\;\mathrm{Solution}\;\Delta_{\mathrm{sol}}{\bar{H}}_{\mathrm{Mg}\left(l\right)}^{\infty }(\mathrm{kJ}/\mathrm{mol})$
1	0.0225	0.16	21.4	−8.6
2	0.0397	0.45	21.2	−8.8
3	0.0276	0.65	21.6	−8.5
4	0.0414	0.95	21.7	−8.3
Average	-	-	21.5	−8.6
Standard error	-	-	1.1	1.1

**Table 4:** Values of the limiting partial enthalpy of the solution of liquid Pd $\Delta_{\mathrm{sol}}{\bar{H}}_{\mathrm{Pd}\left(l\right)}^{\infty }$ in liquid Al. Atmosphere: argon at a pressure *p* = 0.1 MPa; a calibration constant *K* = 0.000003207 kJ/μVs; enthalpy of the pure Pd $\Delta H_{\mathrm{Pd}}^{T_{298}\to T_{1033}}$ = 32.3062 kJ/mol; temperature of the Al bath *T_M_* = 1033 K; and drop temperature *T_D_* = 298 K.

Measurement No.	Dropped Amount of Samples (g)	At.% of Pd in Al Bath	Heat Effects Δ*H*^ef^ (kJ/mol)	$\mathrm{Limiting}\;\mathrm{Partial}\;\mathrm{Enthalpy}\;\mathrm{of}\;\mathrm{Solution}\;\Delta_{\mathrm{sol}}{\bar{H}}_{\mathrm{Pd}\left(l\right)}^{\infty }\;(\mathrm{kJ}/\mathrm{mol})$
1	0.0822	0.14	−154.6	−186.9
2	0.0850	0.28	−154.1	−186.4
3	0.0860	0.42	−154.4	−186.7
4	0.0894	0.57	−154.7	−187.0
5	0.0861	0.71	−154.6	−186.9
Average	-	-	−154.5	−186.8
Standard deviation	-	-	1.1	1.1

**Table 5:** Heat effects Δ*H*^ef^ of the solution and formation enthalpies Δ_f_*H* of the intermetallic phases from the Mg-Pd system. The temperature of the Al bath was 1033 K.

Alloys	T (K)	Sample No.	Δ*H*^ef^ (kJ/mol of atoms)	Δ_f_*H* (kJ/mol of atoms)
14.6 at.% Pd (Mg_6_Pd)	298	1	24.8	−29.0
		2	23.4	−27.6
		3	22.7	−27.0
		4	23.4	−27.7
		5	25.3	−29.5
		6	22.9	−27.2
		Average	23.8	−28.0
		Standard error	1.2	1.2
19.4 at.% Pd ~(ε)	298	1	19.3	−31.9
		2	21.4	−34.1
		3	19.2	−31.9
		4	20.0	−32.7
		Average	20.0	−32.6
		Standard error	1.6	1.6
29.3 at.% Pd ~(Mg_5_Pd_2_)	298	1	17.6	−47.7
		2	16.4	−46.5
		3	16.7	−46.8
		4	16.3	−46.4
		Average	16.7	−46.8
		Standard error	1.4	1.4
35.5 at.% Pd ~(Mg_2_Pd)	298	1	15.5	−56.5
		2	16.1	−57.1
		3	14.6	−55.6
		4	13.8	−54.9
		Average	15.0	−56.0
		Standard error	1.6	1.6

## Data Availability

The data that support the findings of this study are available from the corresponding author, [A.D.], upon reasonable request.

## Supplementary Materials

The following are available online at https://www.mdpi.com/1996-1944/14/3/680/s1, Figure S1. X-Ray diffraction pattern (Co anode ʎ = 1.78 Å) and SEM (BSE) image of Alloy 1. Figure S2. X-Ray diffraction pattern (Co anode ʎ = 1.78 Å) and SEM (BSE) image of Alloy 2. Figure S3. X-Ray diffraction pattern (Co anode ʎ = 1.78 Å) and SEM (BSE) image of Alloy 3. Figure S4. X-Ray diffraction pattern (Co anode ʎ = 1.78 Å) and SEM (BSE) image of Alloy 4.
