# Supplementary material for: Calorimetric Studies of Magnesium-Rich Mg-Pd Alloys
Source: Materials (Basel). 2021 Feb 2;14(3):680. doi: 10.3390/ma14030680 (PMC7867242; doi:10.3390/ma14030680)
Supplement: Supplementary file 1 [file materials-14-00680-s001.pdf]

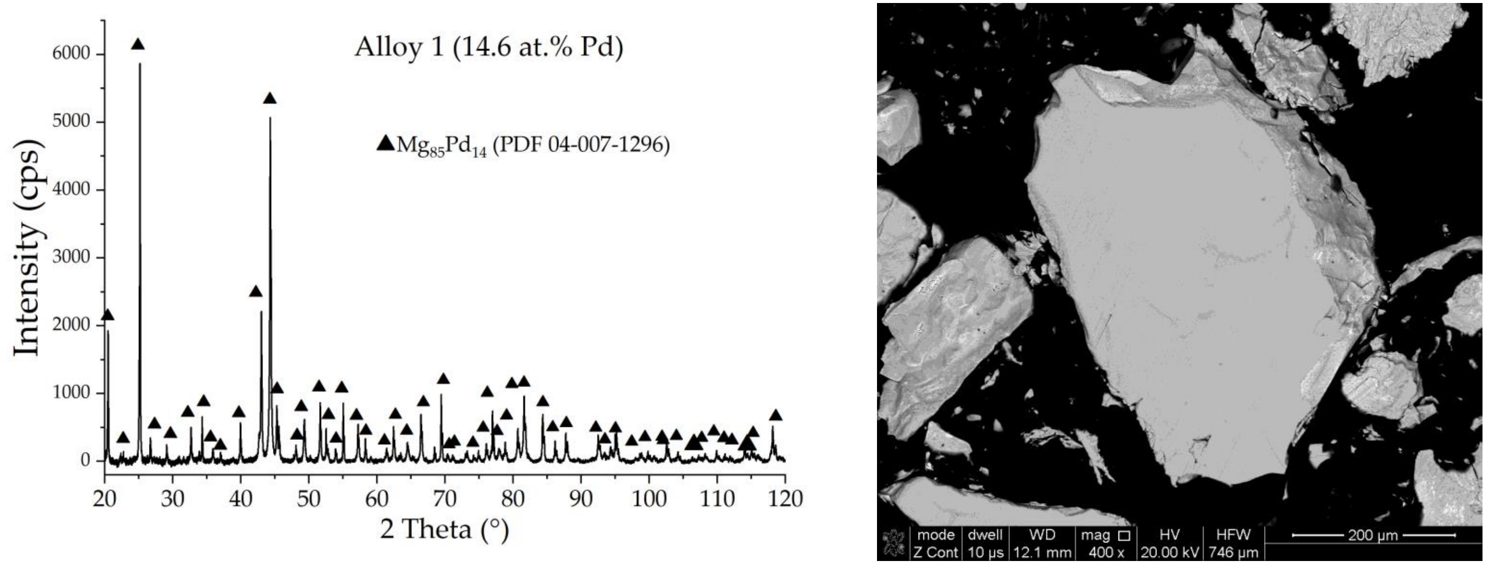

Figure S1. X- Ray diffraction pattern (Co anode λ=1.78 Å) and SEM (BSE) image of Alloy 1 (based on data from [1]).

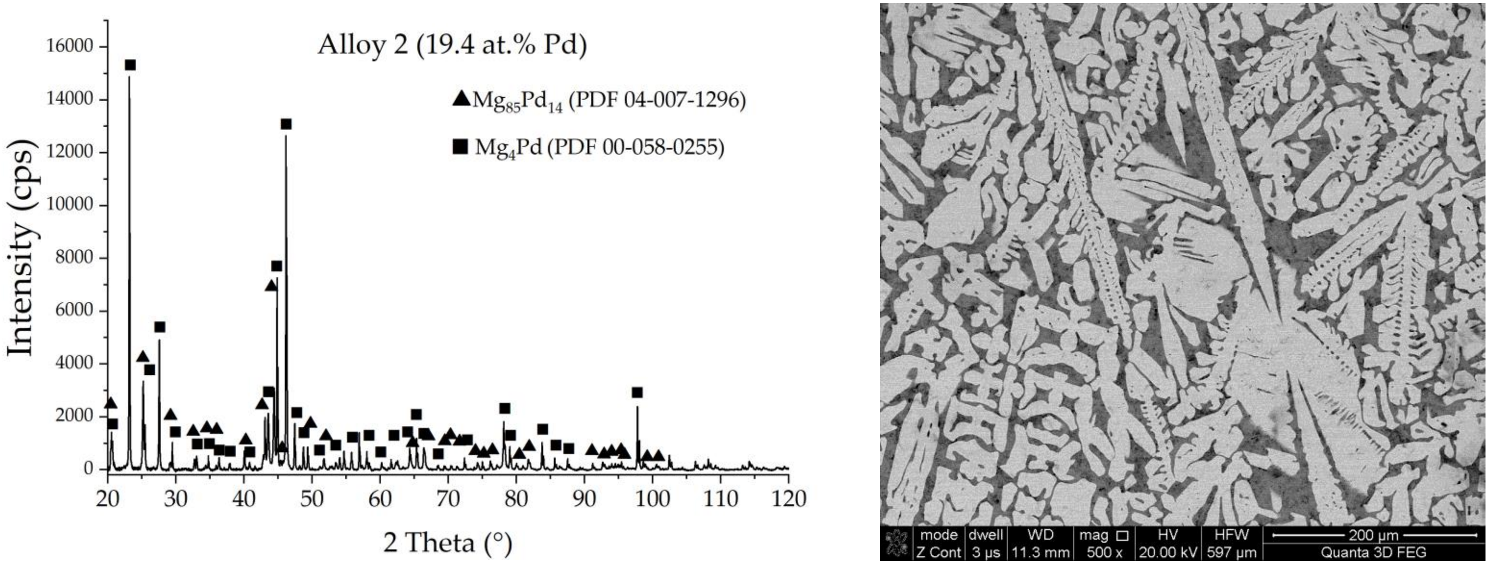

Figure S2. X- Ray diffraction pattern (Co anode λ=1.78 Å) and SEM (BSE) image of Alloy 2 (based on data from [1]).

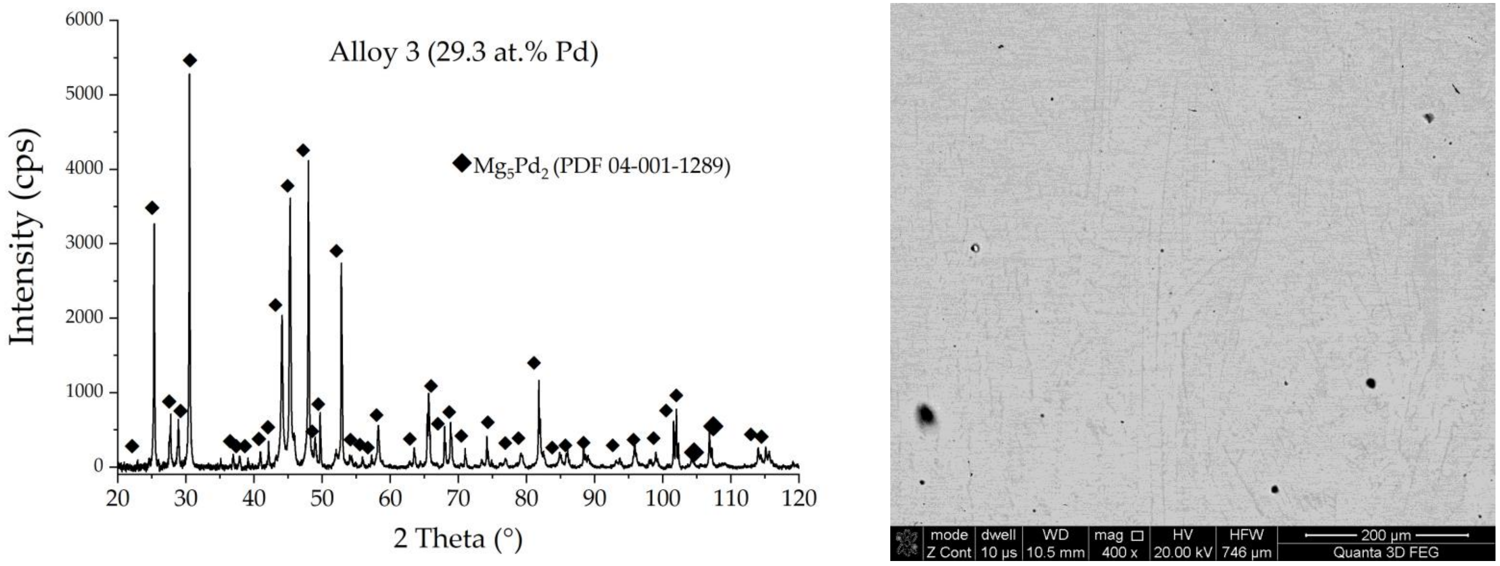

Figure S3. X- Ray diffraction pattern (Co anode λ=1.78 Å) and SEM (BSE) image of Alloy 3 (based on data from [1])..

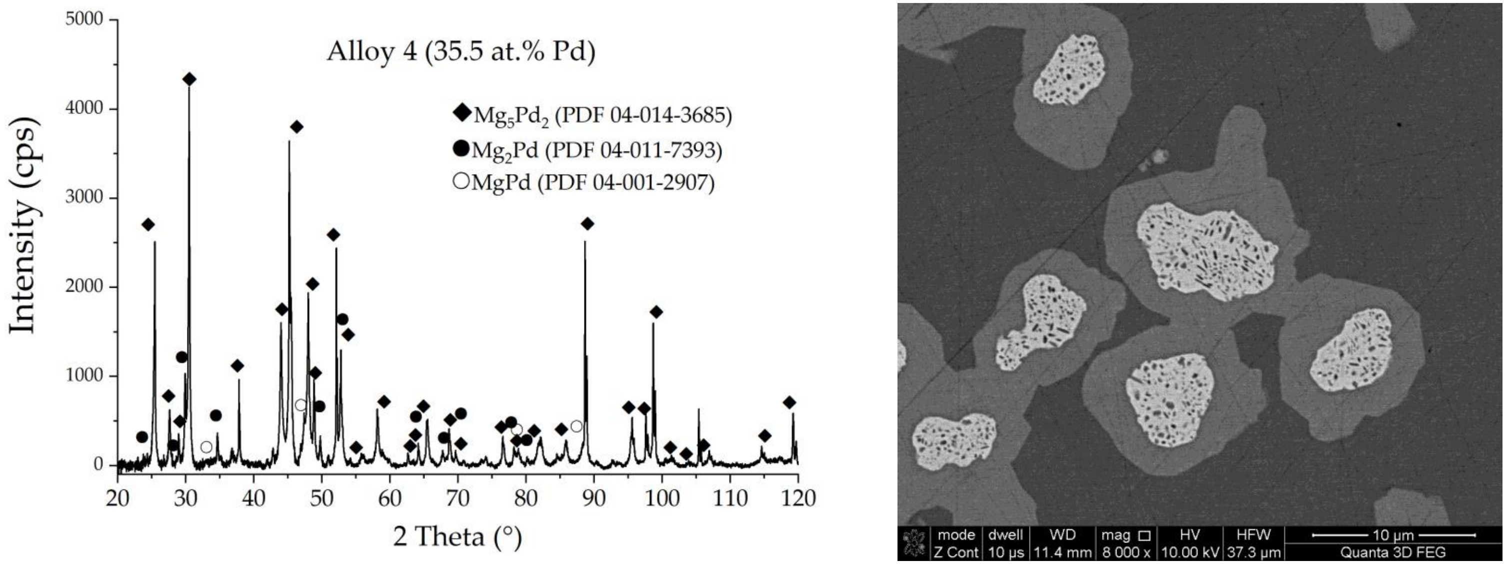

Figure S4. X- Ray diffraction pattern (Co anode λ=1.78 Å) and SEM (BSE) image of Alloy 4 (based on data from [1]).

References

1. Dębski, A.; Pęska, M.; Dworecka-Wójcik, J.; Terlicka, S.; Gąsior, W.; Gierlotka, W.; Polański, M. Structural and calorimetric studies of magnesium-rich Mg-Pd alloys. *Journal of Alloys and Compounds* **2021**, *858*, doi:10.1016/j.jallcom.2020.158085.
